# BCR and chemokine responses upon anti-IgM and anti-IgD stimulation in chronic lymphocytic leukaemia

**DOI:** 10.1007/s00277-016-2788-6

**Published:** 2016-08-20

**Authors:** Andrea Haerzschel, Julie Catusse, Evelyn Hutterer, Manuela Paunovic, Katja Zirlik, Hermann Eibel, Peter W. Krenn, Tanja N. Hartmann, Meike Burger

**Affiliations:** 1 Freiburg University Medical Center, Department of Hematology and Oncology, Albert-Ludwigs-University Freiburg, Breisgau, Germany; 2 Laboratory for Immunological and Molecular Cancer Research, Third Medical Department with Hematology, Medical Oncology, Hemostaseology, Infectious Diseases, and Rheumatology, Oncologic Center, Paracelsus Medical University, Salzburg, Austria; 3 Salzburg Cancer Research Institute, Salzburg, Austria; 4 Center for Chronic Immunodeficiency (CCI), University Medical Center and University Freiburg, Freiburg, Germany; 5 Division of Rheumatology and Clinical Immunology, University Medical Center Freiburg, Freiburg, Germany; 6 Faculty Medical and Life Sciences, Campus Villingen-Schwenningen, University Furtwangen, Schwarzwald, Germany

**Keywords:** CLL, IgM, IgD, BCR signalling, Chemokines

## Abstract

Dysregulation of B cell receptor (BCR) signalling is a hallmark of chronic lymphocytic leukaemia (CLL) pathology, and targeting BCR pathway kinases has brought great therapeutic advances. Activation of the BCR in lymphoid organs has been associated with CLL cell proliferation and survival, leading to progressive disease. While these responses are mediated predominantly by IgM, the role of IgD is less clear. Seeking to uncover downstream consequences of individual and combined stimulation of the two BCR isotypes, we found an amplification of IgD expression and IgD-mediated calcium signalling by previous stimulation of IgM in CLL. Furthermore, no heterologous downmodulation of the isotypes, as observed in healthy donors, was present. Only marginal downregulation of the expression of various chemokine receptors by α-IgM and α-IgD stimulation was found as compared to normal B cells. Consistently, calcium responses of CLL cells to different chemokines were only weakly affected by preceding BCR activation. In contrast, migration towards the two homeostatic chemokines CXCL12 and CCL21 was differentially regulated by IgM and IgD. While IgM activation reduced migration of CLL cells towards CXCL12, but not CCL21, IgD activation predominantly impacted on CCL21 but not CXCL12-mediated chemotaxis. This indicates that the preference for one chemokine over the other may depend on the functional presence of the two isotypes in CLL. Inhibitors against the kinases Syk, Lyn, and Btk antagonised both BCR- and chemokine-induced calcium signals.

**Electronic supplementary material:**

The online version of this article (doi:10.1007/s00277-016-2788-6) contains supplementary material, which is available to authorized users.

## Introduction

Chronic lymphocytic leukaemia (CLL) is characterised by the progressive accumulation of malignant monoclonal B lymphocytes in blood and primary and secondary lymphoid organs. CLL cells are phenotypically mature B cells [1] usually expressing both IgM and IgD on their surface. CLL cells display various degrees of anergy, linked to reduced IgM expression and signalling capacity [2, 3]. High B cell receptor (BCR) signalling capacity in response to IgM [4] as well as IgD [5] stimulation has been associated with adverse prognosis and progressive disease. However, little is known about the functional differences between these two isotypes, and their combined role in CLL.

The importance of antigen stimulation for CLL is further supported by the biased IGHV gene usage in CLL [6, 7]. Unmutated CLL (u-CLL) tend to express low affinity poly- and autoreactive BCRs, while high affinity monoreactive BCRs occur mainly in mutated CLL (m-CLL) and have been shown in certain cases to be specific for bacterial [8] or fungal pathogens [9, 10]. Autonomous BCR signalling was described as a hallmark of CLL [11]. Recently, on a basis of transgenic murine CLL models, it was suggested that both cell autonomous and cell external low affinity BCR interactions contribute to CLL pathogenesis [12]. This obvious reliance of CLL cells on both ligand-dependent and ligand-independent BCR signals has led to the establishment of inhibitors against BCR pathway kinases, targeting Syk, Btk and Lyn for CLL treatment, with great clinical success [13–16]. A prominent effect of Ibrutinib and other inhibitors in vivo is a lymphocytosis caused by the redistribution of CLL cells from lymphoid organs into the periphery [17], based on antagonisation of migration and retention signals [18]. Consistently, chemokine-mediated migration and integrin activation of CLL cells were efficiently inhibited in vitro by targeting Syk [19, 20] and Btk [21]. Collectively, this indicates that the effectiveness of these novel agents is, at least in part, due to a block in the interaction of CLL cells with protective signals from their direct lymphoid microenvironment. The chemokine receptor CXCR4 and its ligand CXCL12 are of particular significance for CLL cell migration and survival [22]. CXCL12 stimulation leads to phosphorylation of Syk [19] and Btk [23]. Other chemokine receptors robustly expressed on CLL cells are CXCR5, CCR7, and the atypical chemokine receptor CCRL2, a presumed regulator of CCR7 activity [24]. Furthermore, CXCR3 is expressed on CLL in variable amounts, in contrast to other B cell lymphomas. High CXCR3 expression levels are associated with indolent disease, exerting a negative functional regulation on CXCR4 [25]. In healthy B cells, activation of the BCR fundamentally alters the expression and function of CXCR4 [26], CCR7, and CXCR5 [27]. In CLL, downregulation of CXCR4 [28] and CXCR5 [29] was observed predominantly in high-risk cases after stimulation with immobilised α-IgM antibodies. In contrast, the impact of IgD activation on the expression pattern of these receptors has not been evaluated yet.

In this study, we examined how IgM and IgD cross-respond to stimulation in CLL and how BCR stimulation impacts on chemokine receptor expression and function, also in the context of therapeutic BCR inhibition.

## Material and methods

### Patient samples and cell isolation

Blood samples were collected from CLL patients after informed consent and ethical approval at the Freiburg Medical University Center and the Third Medical Department, Paracelsus Medical University Salzburg. The samples collected in Salzburg were used for the chemotaxis assays during revision of the manuscript; for all other experiments, the Freiburg cohort was used. A summary of all patients used including IGHV mutation status and BCR expression is given in Supplementary Table 1. Peripheral blood mononuclear cells (PBMCs) from CLL patients and healthy donors were isolated by density gradient centrifugation over Ficoll-Hypaque (Pharmacia, Uppsala, Sweden). Samples were cytometrically analysed for the quantity of CLL cells and included in this study if more than 85 % of cells were CD19 positive.

### Reagents

Murine monoclonal antibody against CCR7 and CCRL2 and PE-labelled antibodies against CXCR5 and CXCR3 were purchased from R&D Systems. PE-labelled antibodies against CXCR4, PE and FITC-labelled antibodies against CD19, and FITC and PE-labelled antibodies against IgD were purchased from BD. PE-labelled antibodies against IgM were purchased from Biolegend and Beckman Coulter. PE-labelled rabbit α-mouse IgG was obtained from Dako Cytomation. Goat F(ab’)_2_ anti-human IgM (α-IgM) and IgD (α-IgD) for stimulations and F(ab’)_2_ of irrelevant specificity as a control were purchased from Southern Biotech. Chemokines were purchased from Peprotech and R&D. Ibrutinib (PCI-32765) was bought from Selleckchem, Bafetinib (INNO 406) from Adooq, and R406 from Riegel Pharmaceuticals. Annexin V-FITC and 7-aminoactinomycin D (7AAD) for determination of cell viability were from Beckman Coulter.

### BCR stimulation

CLL cells and healthy donor PBMCs were thawed and incubated at 37 °C overnight before use. BCR stimulation was performed in 48-well plates. Cells were suspended at 4 × 10^6^ cells per ml, 250 μl of the suspension were applied per well, and α-Ig F(ab’)_2_ was added at a concentration of 20 μg/ml. Immobilisation of the F(ab’)_2_ in wells was performed by dilution in PBS and incubation over night at room temperature. The cell suspension was subsequently added after extensive washing with PBS and RPMI.

### Flow cytometric detection of chemokine receptors and BCR expression

A total of 2.5 × 10^5^ cells were stained with the appropriate antibody for 30 min at 4 °C in PBS containing 0.5 % BSA. Prior to adding the secondary antibody, excess antibody was removed by washing. A minimum of 2 × 10^4^ cells were measured by flow cytometry on a FACSCalibur. Viable cells were identified by SSC-FSC, and the geometric mean of fluorescence intensities (MFI) was determined using FlowJo analysis software. Mean fluorescence intensity ratios (MFIR) were calculated using the appropriate isotypes.

### Intracellular calcium measurement

For measurement of intracellular calcium mobilisation, cells were prepared as described in Quiroga et al. [20]. A total of 1 × 10^7^ cells were incubated in 1 ml complete RPMI with 4 μM Fluo-3-AM (Invitrogen) at 37 °C for 30 min. Cells were then resuspended at 5 × 10^6^/ml and incubated for another 10 min at 37 °C. After washing in complete RPMI, cells were resuspended in medium containing 1.5 mM CaCl_2_ and in case of the inhibitor studies R406, Bafetinib, or Ibrutinib at a concentration of 5 μM. After an incubation of 30 min at 37°, the cells were put on ice. Five minutes before each measurement, 100 μl of cell suspension was added into 400 μl of prewarmed RPMI with CaCl_2_ and, where applicable, with an inhibitor. After 15 s of baseline acquisition, α-IgM F(ab’)_2_, α-IgD F(ab’)_2_ (10 μg/ml), CXCL13 (500 ng/ml), CCL19, CCL21, or CXCL12 (200 ng/ml) were added and the fluorescence intensity was recorded for 2 min. For quantification, the baseline fluorescence intensity was subtracted from the peak intensity after stimulation. The resulting value was termed “calcium response”.

### Chemotaxis

CLL cells were, after 24 h of BCR stimulation, transferred into transwell inserts (Corning Costar) with 5-μm pores. Either medium alone or medium containing CXCL12 (100 ng/ml) or CCL21 (200 ng/ml) was added to the lower well, and the cells were allowed to migrate for 2 h at 37 °C. Migrated cells were then stained for CD5/CD19 and counted using Flow Count Fluorospheres (Beckmann Coulter) as a reference.

### Statistical analysis

Calculation of statistical significance was done using Graph Pad Prism Version 5.03. After assessing the datasets for normal distribution, significances were analysed using paired *t* test in case of normally distributed samples, and Wilcoxon matched pairs test in not normally distributed samples. Differences were considered significant with *p* < 0.05. *P* < 0.05 is marked as *, *p* < 0.01 as **, and *p* < 0.001 as ***.

## Results

### IgM stimulation of CLL cells results in increased calcium mobilisation in response to IgD

**Fig. 1:**
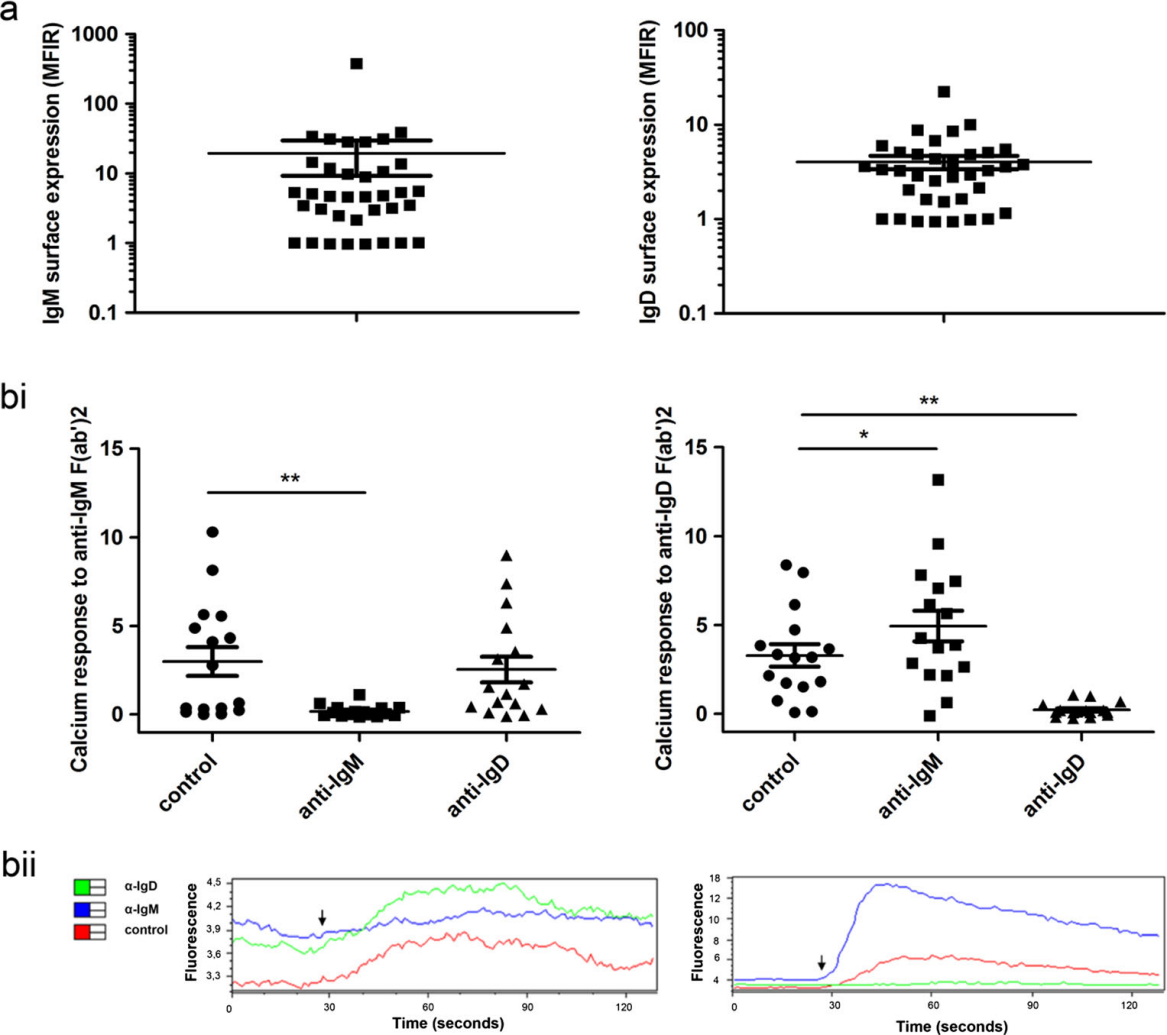
IgM- and IgD-mediated calcium mobilisation in CLL. **a** Surface expression of IgM and IgD was determined by flow cytometry (*n* = 36). A logarithmic scale is used due to the high inter-patient variation. **b** CLL cells were prestimulated by α-IgM or α-IgD antibodies (20 μg/ml) for 24 h, and calcium mobilisation upon a second BCR stimulation was measured by flow cytometry (*n* = 16). Exemplary fluorescence courses are given in *bii*. The *arrows* indicate the time of stimulation

We investigated BCR and chemokine responses upon α-IgM and α-IgD stimulation in peripheral blood CLL samples. Analysing IgM and IgD surface expression in a cohort of 36 samples, we found a considerable inter-patient variability. Except for eight samples harbouring negligible levels of IgM and IgD on their surface (MFIR = 1), all other samples displayed clearly detectable IgM and IgD expressions (Fig. 1a). We did not observe any association of IgM/IgD expression and the IGHV mutational status in this cohort (data not shown).

In functional studies, we first evaluated the impact of IgM or IgD stimulation on further BCR-mediated calcium mobilisation in CLL samples expressing IgM and IgD. In agreement with the findings of Mockridge et al. [2], calcium responses to IgM and IgD stimulation were, like BCR surface expression, highly variable. Fifty-three percent of investigated CLL cases showed a very weak or no calcium response to α-IgM treatment despite detectable surface IgM levels (Fig. 1b, unstimulated controls). In contrast, the overall response of CLL cells to stimulation with α-IgD was higher, with only 12 % of cases displaying no calcium flux. As expected, preincubation with α-IgM or α-IgD resulted in desensitisation of the prestimulated isotype and thus an abolishment of further BCR-triggered calcium releases. However, we did not observe any cross-desensitisation of the other isotypes by IgM or IgD pre-stimulation. In contrast, the response invoked by α-IgD administration was significantly reinforced by previous incubation with α-IgM.

### IgM stimulation increases IgD expression in CLL but not healthy donor-derived B cells

**Fig. 2:**
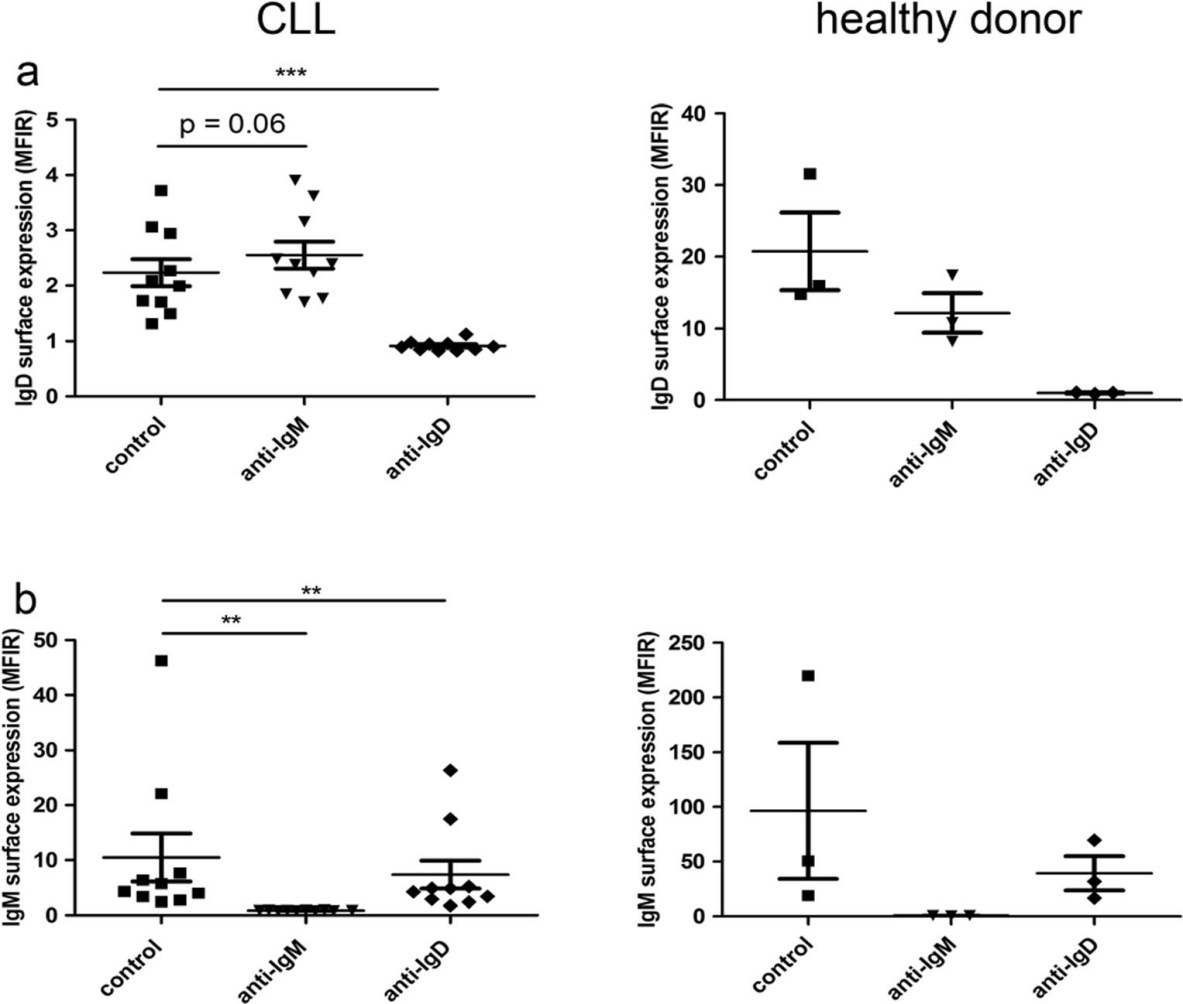
Modulation of BCR surface expression by BCR activation. **a** IgD and **b** IgM surface expression was measured by flow cytometry upon BCR activation by 20 μg/ml α-IgM and α-IgD antibodies for 24 h, compared to the appropriate negative control F(ab’)_2_ in CLL (*n* = 10) and healthy B cells (*n* = 3)

The observation of increased IgD-mediated calcium mobilisation after IgM stimulation raised the question whether this was caused by modulation of IgD surface expression. Indeed, increased IgD-mediated calcium mobilisation in CLL cells upon stimulation with α-IgM was paralleled by a slight increase in IgD surface expression, while a cross-desensitisation was observed in healthy B cells, with a reduction of IgD surface expression after IgM stimulation (Fig. 2a). In contrast, IgD stimulation reduced IgM expression levels in CLL as well as healthy B cells (Fig. 2b).

### Regulation of chemokine receptor surface expression and chemokine-induced calcium responses by α-IgM and α-IgD stimulation is impaired in CLL

The regulation of chemokine receptors is an important process involved in B cell development and activation. Cases of aggressive CLL are characterised by the massive infiltration of bone marrow and lymphoid organs, and altered regulation of homing receptors is involved in disease progression [30]. Therefore, we systematically compared IgM- and IgD-mediated alterations in chemokine receptor expression in CLL and normal B cells.

**Fig. 3:**
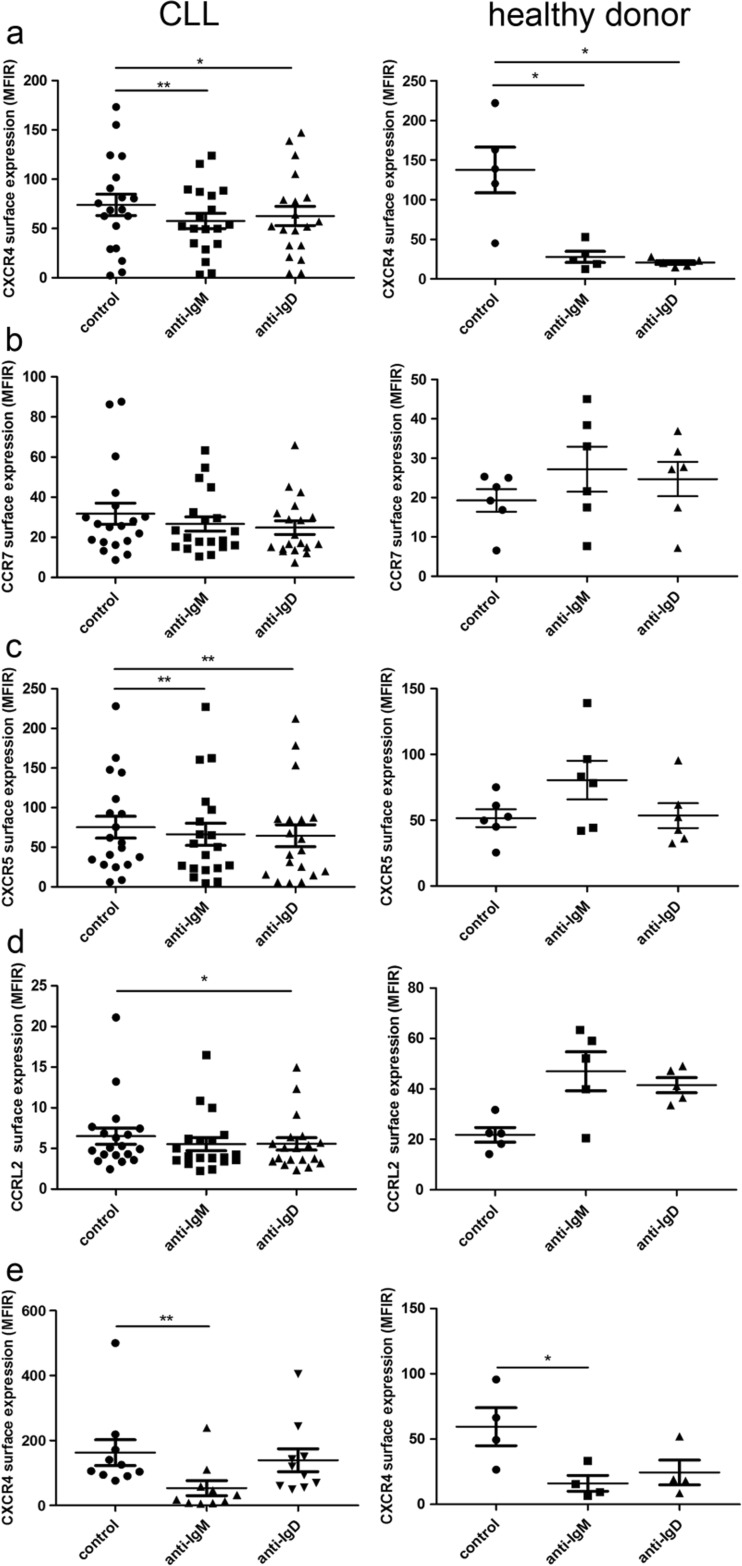
Regulation of chemokine receptors by BCR activation. Surface expression of **a** CXCR4, **b** CCR7, **c** CXCR5, and **d** CCRL2 was measured on CLL (*n* = 19) and healthy donor-derived (*n* = 5) B cells after 24-h stimulation with soluble α-IgM and α-IgD (20 μg/ml) antibodies. **e** CXCR4 expression after 24-h stimulation with immobilised α-IgM and α-IgD (20 μg/ml) was assessed on CLL (*n* = 10) and healthy B cells (*n* = 4). In all experiments, controls were incubated for the same amount of time with the appropriate negative control F(ab’)_2_

The extent of downregulation of CXCR4 expression in CLL by both soluble IgM and IgD stimulation (decrease to 78 and 85 % of the basal expression) was substantially lower than that observed in healthy donor-derived B cells (decrease to 20 and 15 % of the basal expression, Fig. 3a). Also, no significant regulation of CCR7 expression by IgM and IgD stimulation was observed (Fig. 3b). CXCR5 expression was significantly reduced upon stimulation of both isotypes in CLL cells but upregulated by IgM stimulation in healthy donor-derived B cells (Fig. 3c). Notably, CCRL2 was upregulated after stimulation of both isotypes in healthy donor-derived B cells (Fig. 3d), but slightly downregulated in CLL. Surface expression of the chemokine receptor CXCR3 and the B cell marker CD19 (as a control) were not changed by stimulation of IgM or IgD on CLL cells under the same conditions (data not shown).

A stronger BCR stimulus is provided by stimulation with immobilised antibodies, and next, we tested whether this could induce a more distinct chemokine receptor regulation. Indeed, stimulation with immobilised α-IgM significantly reduced CXCR4 expression in CLL cells (Fig. 3e). The extent of downregulation was comparable to that in healthy donor-derived B cells (to 33 % of the original expression in CLL cells and to 27 % in healthy B cells). Immobilised α-IgD exerted no significant effect on CXCR4 expression in CLL, while in healthy B cells, stimulation of IgM and IgD resulted in a comparable CXCR4 downregulation. An IgM-induced reduction of expression was also observed for CXCR5 and CXCR3 (data not shown).

**Fig. 4:**
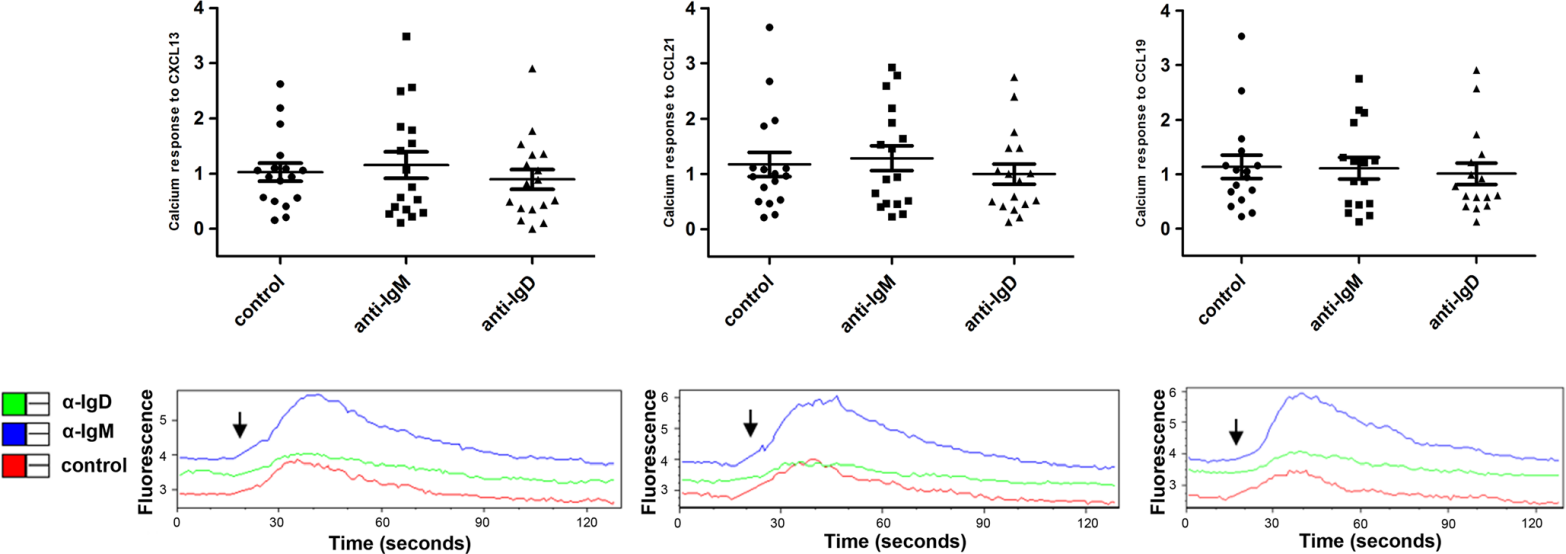
Calcium mobilisation in response to chemokines. CLL cells (*n* = 16) were incubated for 24 h with soluble α-IgM or α-IgD antibodies. Calcium mobilisation was induced by the chemokines CXCL13 (500 ng/ml), CCL21 (200 ng/ml), and CCL19 (200 ng/ml) and determined using the Fluo-3 dye as described in materials and methods. The *lower panels* show representative examples for the kinetics of Fluo-3 fluorescence. The *arrows* indicate the time at which the chemokine was added

To assess the impact of IgM and IgD stimulation on the chemokine receptor system on a more functional basis, we measured the calcium release induced by chemokines. All samples tested showed a significant calcium response to the chemokines CXCL13, CCL19, and CCL21 (Fig. 4). However, this calcium flux was generally lower than that observed after IgM or IgD stimulation. Moreover, the chemokine-induced calcium flux could not be altered by prior IgM or IgD stimulation (Fig. 4).

### Chemotaxis towards CXCL12 and CCL21 is differentially regulated by IgM and IgD activation

**Fig. 5:**
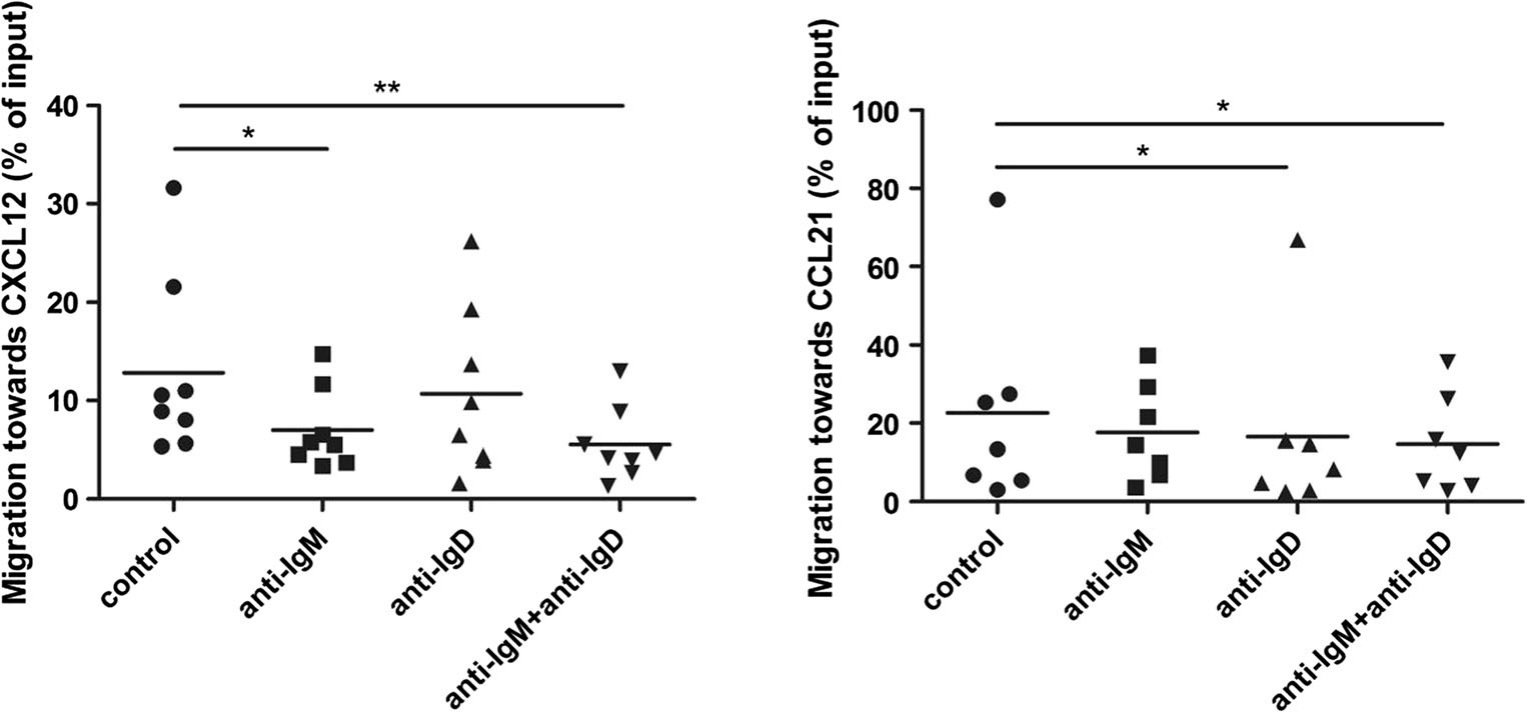
Regulation of chemotaxis by BCR activation. Boyden chamber migration assays were performed towards CXCL12 (100 ng/ml; *n* = 8) and CCL21 (200 ng/ml; *n* = 7) after 24-h stimulation with α-IgM, α-IgD, or both (10 μg/ml). Cells were allowed to migrate for 2 h and subsequently stained for CD19/CD5 and for viability using Annexin-V-FITC and 7AAD. Only Annexin/7AAD double-negative and CD19/CD5 double-positive cells were counted. All experiments were performed as duplicates; results are shown as the percentage of input cells

In contrast to calcium mobilisation, chemotaxis towards CXCL12 was significantly reduced upon IgM stimulation but not affected by IgD stimulation. A double stimulation did not reduce chemotaxis towards CXCL12 beyond the reduction seen after IgM stimulation alone (Fig. 5) and also did not further decrease CXCR4 expression compared to single IgM stimulation (data not shown). In contrast, chemotaxis towards the CCR7 ligand CCL21 was reduced by IgD but not IgM stimulation. A reduction similar to IgD stimulation alone was also observed after IgM/IgD double stimulation.

Taken together, while chemokine receptor regulation in response to stimulation with α-IgM and α-IgD was reduced in CLL, with compensation when using stronger BCR stimulation by immobilised antigens, differential regulation of CXCL12- and CCR7-mediated chemotaxis was observed after IgM and IgD activation.

### Inhibition of Syk, Lyn, and Btk strongly reduces chemokine-induced calcium responses

**Fig. 6:**
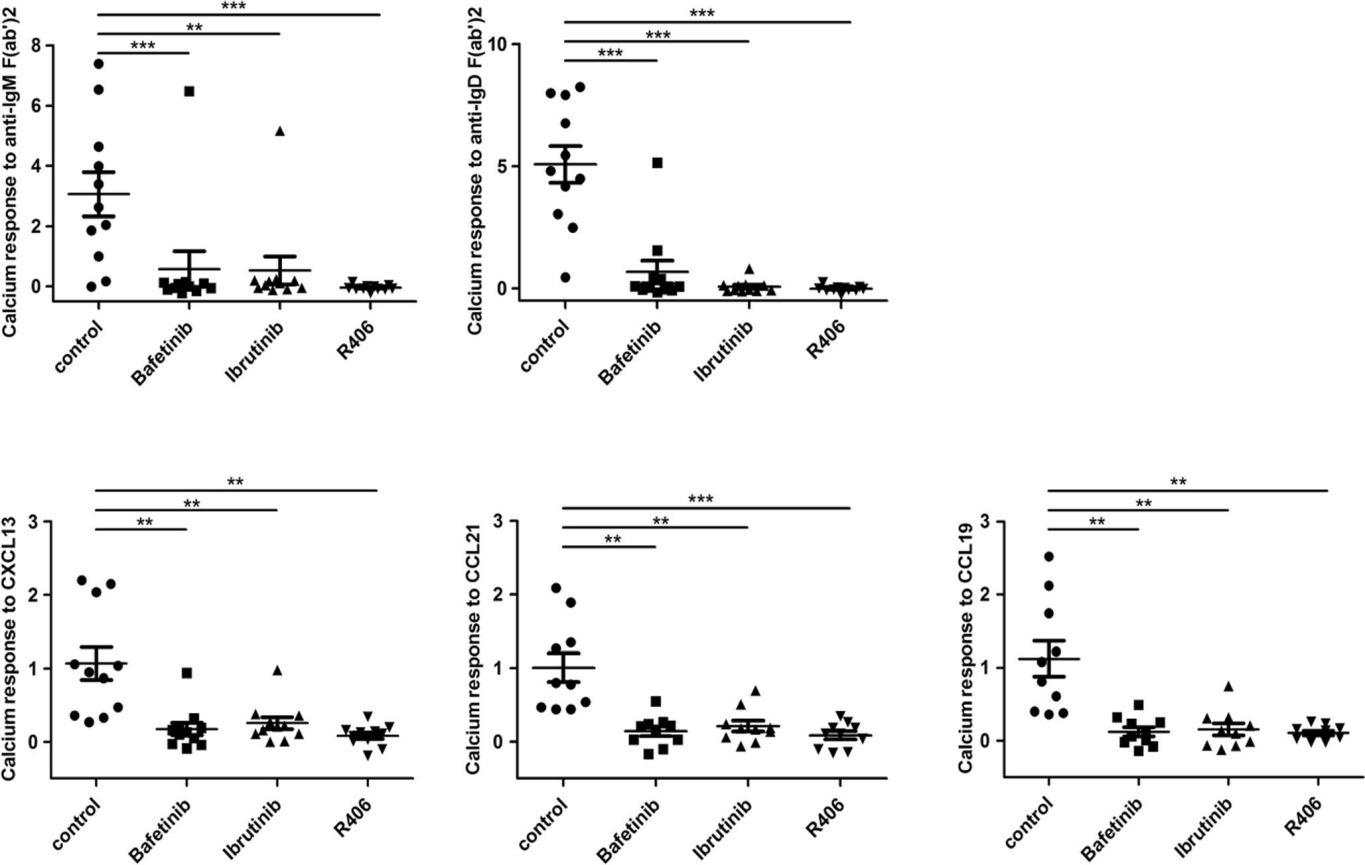
Impact of kinase inhibitors on BCR- and chemokine-mediated calcium mobilisation. Calcium mobilisation of CLL cells in response to α-IgM (10 μg/ml), α-IgD (10 μg/ml), CXCL13 (500 ng/ml), CCL21 (200 ng/ml), and CCL19 (200 ng/ml) was assessed as described. After loading with Fluo-3-AM, cells were additionally incubated for 30 min with 5 μM Bafetinib, Ibrutinib, R406, or an equal volume DMSO as a control (*n* = 11)

Using inhibitors against kinases mediating BCR signalling, remarkable effects on the anatomical localisation of the CLL cells have been observed in clinical trials, possibly by altering chemokine- and integrin-mediated signal transduction. We thus evaluated the impact of the Lyn/Abl inhibitor Bafetinib (INNO-406), the Btk inhibitor Ibrutinib (PCI-32765), and the Syk inhibitor R406 on BCR- and chemokine-mediated calcium mobilisation. Samples of ten patients showing a wide range of BCR-mediated calcium responses were used for this experiment. In all samples, R406 completely abolished BCR-mediated calcium release and strongly reduced the responses to the chemokines CXCL13, CCL19, and CCL21 (Fig. 6). Bafetinib treatment abolished IgM-mediated calcium release in all patient samples but one, and IgD-mediated response in all except for two cases. Responses to all three chemokines tested were strongly diminished, albeit not completely abrogated. Ibrutinib prevented calcium release after IgM and IgD activation in all cases but one (Fig. 6), also at lower concentrations (Supplementary Fig. 1). It also considerably reduced chemokine-mediated calcium mobilisation. Altogether, these therapeutics are highly effective in abrogating calcium mobilisation after BCR as well as chemokine receptor activation in the majority of CLL samples.

## Discussion

CLL is a highly environment-dependent tumour with cells quickly dying when taken into solo cell culture [31]. BCR activation is a central stimulus driving CLL survival, proliferation, and pathogenesis [7]. Most CLL cells coexpress IgM and IgD, yet the ratio is highly variable and patient specific. The significance of this coexpression and individual functions for each isotype in normal and malignant B cells are still poorly understood. We thus aimed to determine their influence onto each other as well as on the chemokine system. Early papers reported heterologous desensitisation of IgD function by IgM prestimulation and vice versa in normal mouse and human B cells [32, 33]. In contrast to these findings in non-leukemic B cells, we found no desensitisation of the heterologous isotype in terms of calcium signalling or surface expression after selective stimulation of one BCR isotype in CLL. Notably, there was even an enforcement of IgD-invoked calcium responses by previous incubation with α-IgM antibodies. This went along with slightly increased IgD expression, while in healthy B cells a clear heterologous downmodulation was observed. Similar observations have previously been made in murine lymphoma cells [34], indicating that this phenomenon may be intrinsic to certain types of B cell malignancies. Taking into account the autonomous BCR signalling in CLL, the increase in calcium mobilisation and surface expression may reflect the inhibition of a constitutive internalisation and recycling process by IgM stimulation, leading to an accumulation of IgD at the cell surface.

BCR activation fundamentally alters the expression of several chemokine receptors on normal B cells in vivo, thereby assuring their correct localisation during the following steps of the immune response [35]. Our results confirm the previous observation of CXCR4 downmodulation in normal B cells upon IgM engagement [26] and add the observation of a comparable reduction upon IgD activation in these cells. In contrast, CLL cells were less susceptible to IgM- and IgD-induced CXCR4 and CXCR5 regulation than normal B cells, which may mirror the disrupted architecture and diffusion of the regular follicular chemokine gradients in CLL lymphoid organs [36, 37]. Notably, the impaired chemokine receptor regulation upon weak soluble BCR stimulation was independent of the extent of general IgM responsiveness and thus not attributable to general features of anergy. It was also uncoupled from functional regulation as we observed comparable effects to the reduction in migration by soluble stimulation to those reported by Vlad [28] using immobilised BCR stimulation.

An interesting novel finding of our study is the differential influence of IgM versus IgD activation on the chemotactic preference of CLL cells. IgM-stimulated CLL cells retained chemotaxis towards the key lymph node chemokine CCL21 but displayed downregulated migration to CXCL12 suggesting a preferential role of CCR7 for migration of antigen-stimulated CLL cells within the lymphoid microenvironment. In contrast, the retained chemotaxis towards CXCL12 but not CCL21 upon IgD stimulation may suggest a different niche preference of these cells. Further studies should address whether the homing propensities of CLL cells to distinct organs depend on different IgD/IgM ratios of these cells.

Finally, we found inhibition of both chemokine- and BCR-mediated calcium responses by R406, Bafetinib, and Ibrutinib. While expected for BCR-induced calcium signalling, the strong dependence of chemokine receptor-induced calcium signals for all three kinases was unanticipated. This indicates a largely overlapping signalling cascade leading to the mobilisation of intracellular calcium, which probably converges on the common regulator Plcγ2. Residual Plcγ2 activity upon Lyn or Btk inhibition [38, 39] may account for intact calcium signals in exceptional patient samples (Fig. 6a) and could be caused by activating mutations, which should be addressed in future studies in detail.

Taken together, our data show that in addition to the generally reduced BCR responsiveness, CLL cells display defects in the regulation of chemokine receptors after BCR activation, as well as a specific regulation of migratory preferences by IgM and IgD.

## Electronic supplementary material

Below is the link to the electronic supplementary material.

Supplementary Table 1 Characterisation of patient samples used for this study. IGHV mutation status (M = mutated; UM = unmutated; ND = not determined), the cohort (FR = Freiburg; S = Salzburg) and IgM and IgD surface expression as MFIR measured 2 h after thawing are summed up for all patient samples used in this study. All patients were untreated at the time of sample collection. Measurements of the Freiburg cohort were performed using α-IgM-PE antibody from Biolegend and α-IgD-FITC antibody from BD, measurements of the Salzburg cohort using α-IgM-PE antibody from Beckman Coulter and α-IgD-PE antibody from BD. (PDF 122 kb)

Supplementary Fig. 1 Effect of 1 μM Ibrutinib on BCR mediated calcium mobilisation. Calcium mobilisation of CLL cells in response to α-IgM (10 μg/ml), α-IgD (10 μg/ml) after incubation with 1 μM Ibrutinib was assessed (n = 3). Calcium responses after inhibitor treatment were normalised to the control responses. (TIF 27169 kb)
