# Supplementary material for: BCR and chemokine responses upon anti-IgM and anti-IgD stimulation in chronic lymphocytic leukaemia
Source: Ann Hematol. 2016 Aug 20;95(12):1979–88. doi: 10.1007/s00277-016-2788-6 (PMC5093209; doi:10.1007/s00277-016-2788-6)
Supplement: Supplementary file 1 — Characterisation of patient samples used for this study. IGHV mutation status (M = mutated; UM = unmutated; ND = not determined), the cohort (FR = Freiburg; S = Salzburg) and IgM and IgD surface expression as MFIR measured 2 h after thawing are summed up for all patient samples used in this study. All patients were untreated at the time of sample collection. Measurements of the Freiburg cohort were performed using α-IgM-PE antibody from Biolegend and α-IgD-FITC antibody from BD, measurements of the Salzburg cohort using α-IgM-PE antibody from Beckman Coulter and α-IgD-PE antibody from BD. (PDF 122 kb) [file 277_2016_2788_MOESM1_ESM.pdf]

Pat.Nr	IGHV mutation	Cohort	IgM MFIR	IgD MFIR
1	UM	FR	4,6	1,6
2	M	FR	4,6	3,0
3	M	FR	374,6	22.32
4	M	FR	5,1	2,9
5	M	FR	5,6	6,8
6	UM	FR	5,3	3,4
7	M	FR	3,1	5,1
8	UM	FR	13,8	8,6
9	M	FR	1,0	1,0
10	M	FR	31,4	3,6
11	ND	FR	28,5	3,3
12	M	FR	2,2	4,4
13	M	FR	3,2	3,6
14	M	FR	38,9	8,8
15	M	FR	1,0	2,8
16	UM	FR	1,0	1,0
17	M	FR	1,0	1,0
18	UM	FR	4,7	1,2
19	UM	FR	31,6	6,0
20	UM	FR	1,0	1,0
21	UM	FR	11,9	2,5
22	UM	FR	1,0	1,0
23	M	FR	3,5	4,9
24	UM	FR	9,8	2,1
25	UM	FR	2,5	3,3
26	UM	FR	3,0	3,8
27	UM	FR	28,6	2,1
28	M	FR	1,0	1,0
29	M	FR	3,5	4,0
30	M	FR	1,0	1,0
31	M	FR	9,0	1,5
32	UM	FR	34,3	4,9
33	UM	FR	5,4	5,1
34	UM	FR	10,6	5,5
35	UM	FR	14,5	1,6
36	M	FR	4,8	10,0
37	M	S	8,4	11,9
38	UM	S	8,5	9,4
39	UM	S	3,6	4,4
40	UM	S	8,0	26,1
41	M	S	5,2	16,5
42	M	S	5,4	10,9
43	M	S	10,7	28,0
44	UM	S	7,4	5,2
