# Supplementary figures and images for: BCR and chemokine responses upon anti-IgM and anti-IgD stimulation in chronic lymphocytic leukaemia
Source: Ann Hematol. 2016 Aug 20;95(12):1979–88. doi: 10.1007/s00277-016-2788-6 (PMC5093209; doi:10.1007/s00277-016-2788-6)

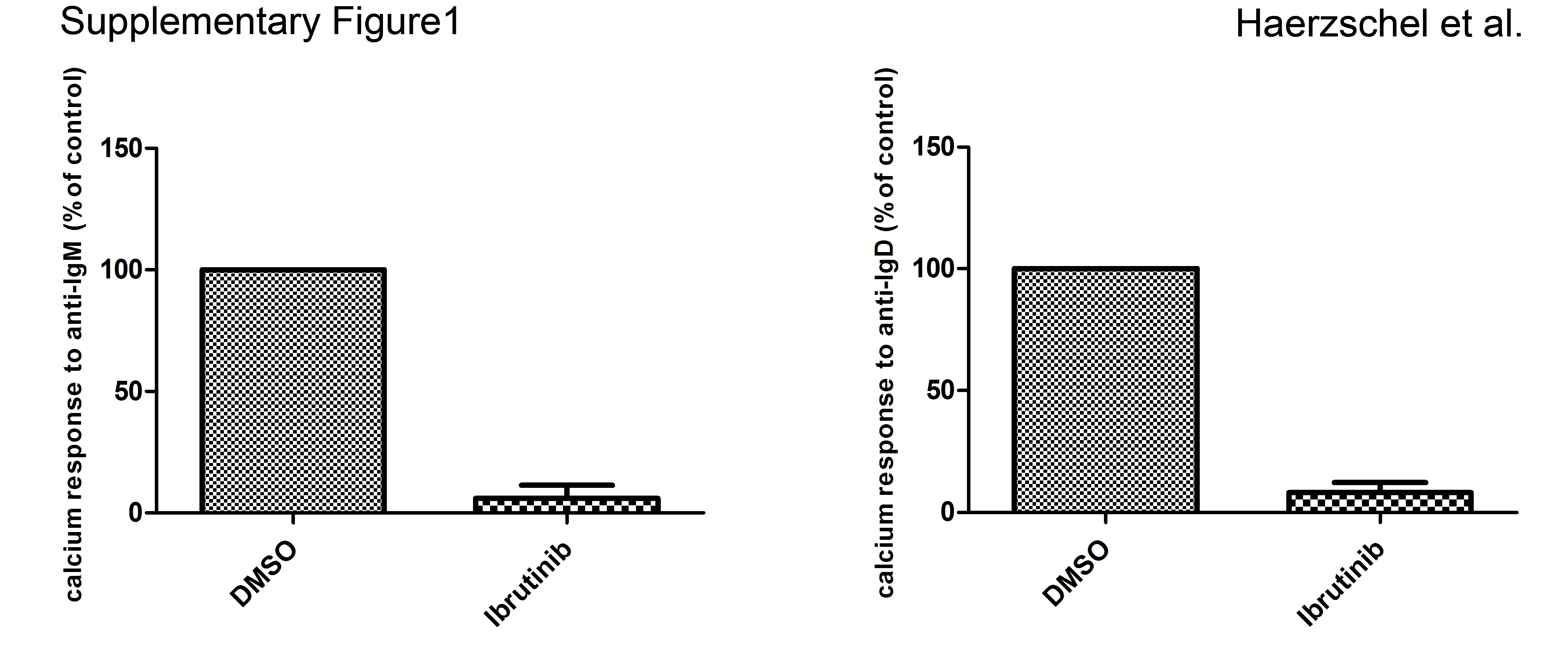

Supplement: Supplementary file 2 — Effect of 1 μM Ibrutinib on BCR mediated calcium mobilisation. Calcium mobilisation of CLL cells in response to α-IgM (10 μg/ml), α-IgD (10 μg/ml) after incubation with 1 μM Ibrutinib was assessed (n = 3). Calcium responses after inhibitor treatment were normalised to the control responses. (TIF 27169 kb) [file 277_2016_2788_MOESM2_ESM.tif]
